# Comparative Evaluation of Compression Testing Methods for Murine Lumbar Vertebral Bodies: Identifying Most Reliable and Reproducible Techniques for Assessing Compressive Strength

**DOI:** 10.3390/bioengineering12030273

**Published:** 2025-03-10

**Authors:** Daniel Kronenberg, Britta Wieskoetter, Sarah Soeger, Heriburg Hidding, Melanie Timmen, Michael J. Raschke, Richard Stange

**Affiliations:** 1 Department of Regenerative Musculoskeletal Medicine, Institute of Musculoskeletal Medicine, University of Muenster, 48149 Muenster, Germany; 2 Department of Orthopaedics, Trauma, Hand and Reconstructive Surgery, University Hospital Münster, Marienhospital Steinfurt, 48565 Steinfurt, Germany; 3 Department of Trauma, Hand and Reconstructive Surgery, University Hospital Muenster, 48149 Muenster, Germany

**Keywords:** compression assay, method comparison, lumbar vertebra, mouse model

## Abstract

This study evaluates four compression testing methods to determine the most reliable and reproducible technique for assessing the compression strength of murine lumbar vertebral bodies. Twenty female C57BL/6 mice (12 weeks old) were randomized into four groups: Group 1, compression of the complete lumbar vertebral body (LVB) with dorsal spinal processes; Group 2, compression at the vertebral body surface; Group 3, compression at the vertebral body surface after vertebral arch resection; Group 4, resection of the vertebral arch with straightening of the intervertebral joint surface. A mono-axial static testing machine applied compression, measuring load to failure, stiffness, yield load, and elasticity modulus. Method 1 resulted in significantly higher load-to-failure and yield-to-failure (25.9 N compared to 18.2 N, and twice 12 N for Methods 2–4), with the least variation in relative values. Method 3 had increased stiffness and a significantly higher Young’s modulus (232 N/mm, in contrast to 101, 130, and 145 N/mm for Methods 1, 2, and 4, respectively) but yielded inconsistent results. Method 4 showed the greatest variability across specimens. Method 2 yields suitable data quality as well, albeit with a slightly higher variation, and is the recommended procedure if the spinal processes have to be excluded from the measurement. Based on these findings, Method 1 produced the most consistent and reproducible data and is recommended for future studies evaluating vertebral biomechanics in mice.

## 1. Introduction

Animal experiments involving mice offer several advantages, including ease of handling, a short reproductive cycle, and low housing costs due to their small size. In 1989, Mario Capecchi introduced a method for manipulating embryonic stem cells, which became the standard for modifying the murine germline. This innovation led to the development of a wide array of mouse models for studying physiological processes and pathological conditions. Consequently, small animal models have become indispensable for investigating the complex mechanisms underlying bone metabolism and healing. Moreover, transgenic mice serve as a valuable tool for analyzing gene function and its impact on disease outcomes [1,2]. Various methods are available to induce osteoporosis in mice, providing robust models for research. To determine the functional differences introduced by these models in bone, biomechanical testing is an important method in experimental studies to evaluate stability, stiffness, and weight bearing capacity [2,3,4]. In a murine model, size poses a significant problem when analyzing the bone strength in biomechanical studies.

Bone tissue consists of two main components: cortical and cancellous (or trabecular) bone, which differ in their composition and mechanical properties. The ratio of these two types of bone varies across the skeleton, contributing to differences in biomechanical properties. Importantly, gene and protein expression can influence cortical and trabecular bone in distinct ways. Studies have shown that certain proteins significantly impact the structure and stability of cortical bone [3,4,5,6,7,8]. Therefore, it is necessary to investigate different skeletal regions to distinguish the effects of genetic and molecular targets on cortical versus cancellous bone. For instance, vertebral bodies have a higher proportion of cancellous bone compared to cortical bone. This characteristic makes the vertebral body, particularly in the lumbar spine, a well-established model for evaluating trabecular bone structure and its changes, as alterations in bone morphology often appear earlier in trabecular bone [7,9,10].

**Figure 1:**
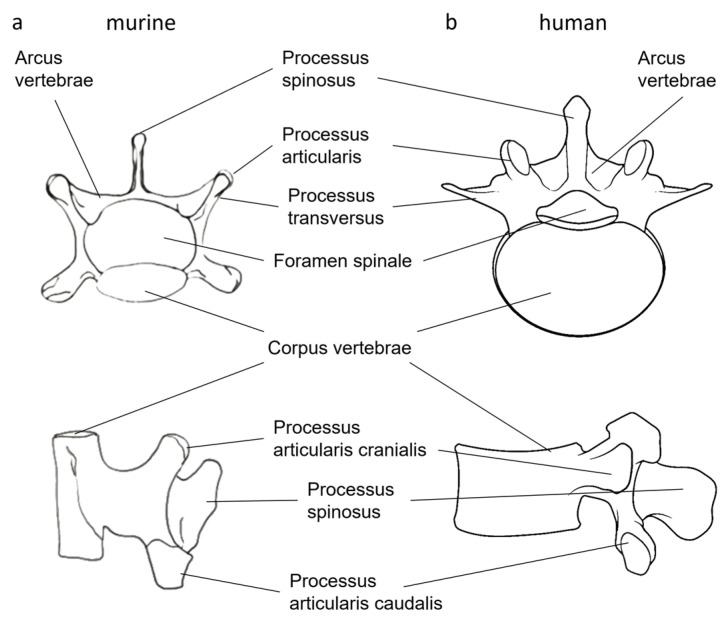
Anatomy of lumbar vertebral body of mouse (**a**) and human (**b**) modified after [11,13].

The murine lumbar spine comprises six vertebral bodies with a basic morphology that shares similarities with the human lumbar spine, including a ventral vertebral body (corpus vertebrae), a dorsal vertebral arch (arcus vertebrae) surrounding the spinal canal, and the lateral transverse processes (processus transversus). The vertebral joints are formed by the cranial and caudal articular processes (processus articularis cranialis et caudalis), with the caudal articular process extending beyond the base of the vertebral body [11] (Figure 1a).

Despite these similarities, notable anatomical differences exist between the mouse and human vertebral bones. In mice, the ratio of the vertebral body to the vertebral arch is smaller, and the morphology is elongated and narrow, likely due to the lack of axial compression. The vertebral arch, which houses the spinal canal, is proportionally larger in mice compared to the vertebral body [12] (Figure 1b).

Since murine vertebral bodies are just some millimeters in size, the biomechanical testing method must be chosen wisely to obtain valid information. A variety of very different compression testing methods for vertebral bodies are published [1,2,4,8,9,14,15]. They differ in manipulation of the bone before measurement due to the bone morphology and embedding into the testing machine. However, the results are difficult to compare. There is no current standardized testing model for vertebral bodies in the literature to date. Although, it becomes even more important due to the numerous murine studies to be able to compare the results of different strains and models to each other.

Compression testing determines the reaction of biological material under load. The tissue properties (stiffness and load to failure) describe the extrinsic property of the individual vertebral body, they are determined both by the properties of the tissue and by the size of the sample. The material property (toughness and Young’s modulus) is consistent with the stability of the material “vertebral body”. The tissue properties are normalized by cross-sectional area and are converted to a stress–strain curve from which the properties of the tissue (intrinsic properties) itself can be determined. The ultimate stress is a measure of the material strength. The slope of the stress–strain curve defines the elastic (Young’s) modulus of the tissue, whereas the area under the stress–strain curve is the modulus of toughness, which is the energy required to cause failure of the bone matrix itself, independent of the sample size.

The aim of this study is to evaluate the reliability and reproducibility of different established compression testing methods for the murine lumbar vertebral bodies and to identify the most appropriate testing method to serve as a standard. We analyzed the performance, precision, and reproducibility of the individual methods on the basis of biomechanical parameters.

Given the small size of the murine vertebral bodies, typically only a few millimeters in diameter, selecting an appropriate compression testing method is crucial to obtain reliable data. A variety of compression testing methods have been published, with differences in sample preparation and the manner in which bones are embedded into the testing apparatus [1,2,4,8,9,14,15]. Either the complete vertebra [8] or only the smaller Corpus vertebrae is tested [12]. Also, manipulation of the bone, such as removing the vertebral arc [1] or straightening the surface by trimming parts of the bone [14], has been recognized. However, the lack of standardization across these methods complicates a comparison of the results. To date, no universally accepted testing model for the murine vertebral bodies has been established. This is particularly significant as numerous murine studies have been conducted, necessitating a standardized approach to enable meaningful comparisons across different strains and experimental models.

Compression testing measures the response of biological material to applied load. Tissue properties, including stiffness and load to failure, reflect the extrinsic properties of individual vertebral bodies, and are influenced by both tissue composition and sample size. Material properties, such as toughness and Young’s modulus, represent the intrinsic stability of the bone tissue itself. Normalizing tissue properties by the cross-sectional area yields stress–strain curves, from which intrinsic material properties can be derived. The ultimate stress measures material strength, the slope of the stress–strain curve defines the elastic (Young’s) modulus, and the area under the curve represents the modulus of toughness, which quantifies the energy required to fracture the bone matrix independently of sample size.

The objective of this study is to evaluate the reliability and reproducibility of various established compression testing methods for the murine lumbar vertebral bodies and to identify the most suitable testing method for standardization. The performance, precision, and reproducibility of individual methods are assessed based on biomechanical parameters.

## 2. Materials and Methods

### 2.1. Specimen Preparation

All animal experimental methods are reported in accordance to the ARRIVE guidelines (https://arriveguidelines.org, accessed on 12 March 2024). All procedures were approved by local government animal rights authorities in accordance with NIH guidelines (Landesamt für Natur, Umwelt und Verbraucherschutz, Nordrhine-Westphalen, Germany G67/2006). All mice used in this study were female C57BL/6 littermates genotyped as wild type (wt), kept in family groups in an air-conditioned room at a constant temperature of 20–23 °C, and fed a standard laboratory diet with ad libitum access to tap water. The animals were sacrificed at 12 weeks by cervical dislocation, with procedures approved by the local government animal rights protection authorities in accordance with NIH guidelines (Landesamt für Natur, Umwelt und Verbraucherschutz, Nordrhine-Westphalen, Germany G67/2006). The fourth lumbar vertebra (L4) was used, dissected, and thoroughly cleaned of soft tissue and the vertebral disk.

**Figure 2:**
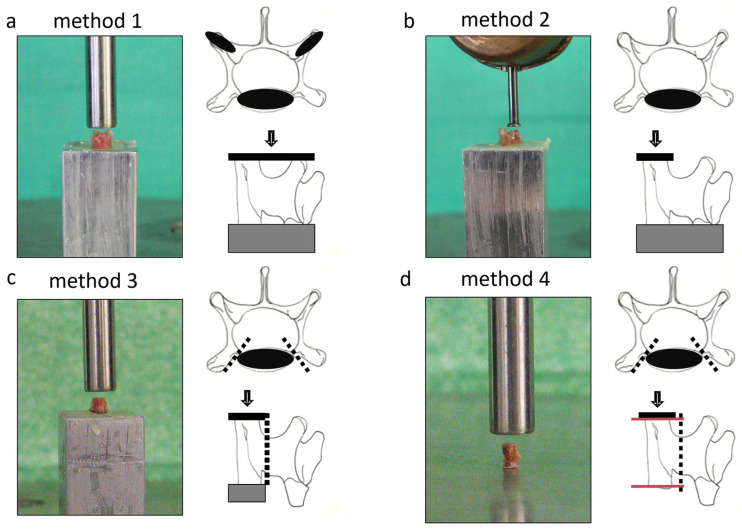
The compression testing of the murine vertebral body (n = 5) with four different testing methods: the black areas present the areas of the applied axial pressure. The dashed lines show the resection of the transversal and dorsal process. The grey area presents the fixation. (**a**) Method 1: the contact area of the testing tool with the LVB included the transversal process and the cranial surface of the body. (**b**) Method 2: the contact area of the testing tool with the LVB excluded the transversal process only providing the pressure on the complete cranial surface of the vertebral body. (**c**) Method 3: the transversal and dorsal process were resected from the vertebral body, and axial pressure was applied on the complete cranial surface of the vertebral body. (**d**) Method 4: axial pressure was applied on the complete cranial surface of the vertebral body.

The vertebral bodies were prepared in four different manners before biomechanical compression testing (Figure 2):

- Method 1: The whole vertebral body was placed upright and fixated without bone manipulation using bone cement (Palacos R, Heraeus Kulzer GmbH, Germany) to provide a planar surface and unidirectional force application. The dorsal process was pressed into the cement until the caudal surface was horizontal.
- Method 2: The placement and fixation were the same as Method 1, but a smaller diameter compression tool was used, excluding the transversal process.
- Method 3: The transversal and dorsal processes were resected, and the remaining vertebral body was fixated into the cement.
- Method 4: Similar to Method 3, but the cranial and caudal surfaces were ground to create parallel surfaces, eliminating the need for resin embedding.

### 2.2. Determination of Dimensional Parameters of the Vertebral Bodies

First, the height of the vertebrae and vertebral bodies as well as their diameters were measured to determine the initial length and initial cross-sectional area for further calculations. In the first method, the cross-sectional area was assumed to be a ring with an elliptical thickening. Additionally, the inner and outer diameters at the caudal end of the vertebral arches were measured, and an average value for the ring thickness was calculated. In Methods 2–4, only the cross-sectional area of the vertebral body was considered elliptical using the following formula:

(1) $$A_{0}=\frac{a}{2}*\frac{b}{2}*\pi$$

where *a* is the diameter at the widest and *b* is the diameter perpendicular to *a*. Every length was obtained using calipers and the mean of five measurements were taken.

### 2.3. Compression Testing

Prior to biomechanical analysis, sample dimensions were determined. The vertebral arc surface area was approximated to a ring-like surface, and the caudal end plate to an ovoid area for calculating Young’s modulus. The change in length as a function of force was used to determine load to yield and load to failure [16]. Values were normalized by setting the mean to 100%.

**Figure 3:**
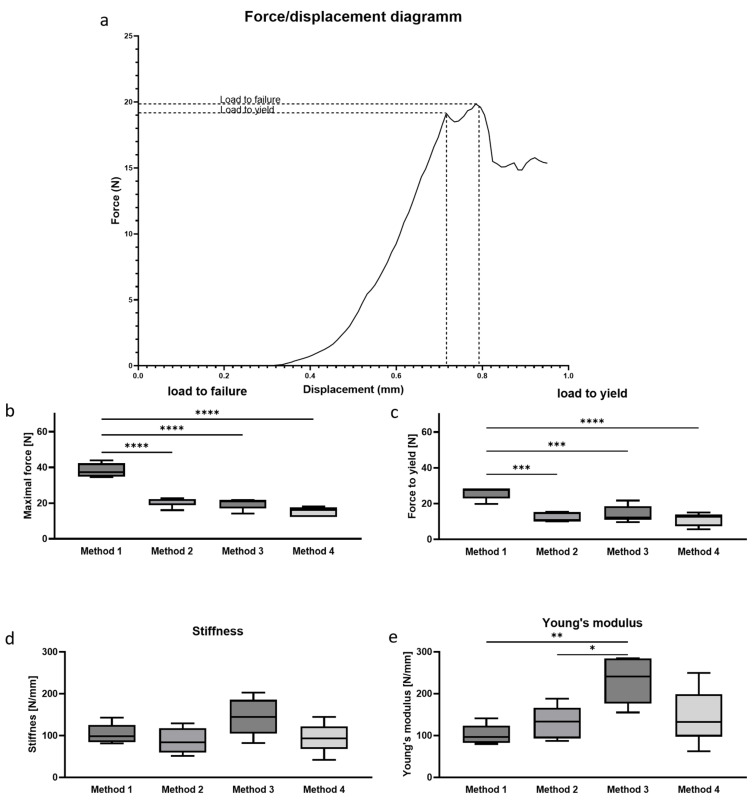
(**a**) Representative force/displacement graph for the read-out of the biomechanical testing. The point of yield (turning point of the slope) and the point of failure (maximum force) are marked with dashed lines. (**b**–**e**) Biomechanical parameters obtained by compression testing the fourth lumbar vertebral body using the four given methods until failure. (**b**) Load to failure in N; (**c**) load to leave the elastic properties of the sample in N; (**d**) stiffness obtained by calculation to the slope in the force/displacement graph at the elastic increment in N/mm; (**e**) Young’s modulus normalizing the stiffness over the surface area to obtain the dimensionless material constant in N/mm. Statistical test: One-way ANOVA followed by Tukey’s multiple comparisons test. The *p*-values were classified as follows: * *p* < 0.05, ** *p* < 0.01, *** *p* < 0.001, and **** *p* < 0.0001.

Compression test was conducted with a compression testing machine (Lloyd LRK5, Lloyd Instruments, Bognor Regis, UK) measuring the applied force at a rate of 8 kHz using a 250 N load cell with 0.5% accuracy at forces over 5 N, with a velocity of 1 mm/min under replacement control. The force–displacement curve was visualized using Nexygen plus 2.1 software (AMETEK Lloyd Instruments). Load to failure was defined as the maximum load point, and yield load was defined as the turning point between elastic and plastic deformation (Figure 3a). Stiffness was calculated from the linear part of the curve, and energy and elasticity moduli were derived from the data. Stiffness is the slope of the linear part of the force/displacement graph [16].

(2) $$s=\frac{F_{f}-F_{0}}{{∆l}_{f}-{∆l}_{0}}$$

Young’s modulus was calculated by using the surface of the bone (*A*). The surface was determined as described above.

(3) $$E=\frac{S*l_{0}}{A}$$

Data were compared to recently described methods for evaluating the stability of the murine lumbar vertebral bodies.

### 2.4. Statistical Analysis

One-way ANOVA followed by Tukey’s multiple comparisons test were performed using GraphPad Prism version 10.0.0 for Windows (GraphPad Software, Boston, MA, USA). The *p*-values were classified as follows: * *p* < 0.05, ** *p* < 0.01, *** *p* < 0.001, and **** *p* < 0.0001. Standard deviation and statistical error were also calculated to assess data variation.

### 2.5. Data Availability

Data is provided within the manuscript or the Supplementary Information files.

## 3. Results

### 3.1. Determination of Absolute Biomechanical Properties

Load to failure: The numerical values decreased with increasing levels of manipulation (Figure 3b) Without any manipulation of the bone (Method 1), the maximum load of the lumbar vertebrae in mice was 38.3 N. With Methods 2 and 3, the values decreased to 20.8 N and 20.3 N, respectively, which is approximately 70% of the result from Method 1. Using the maximal manipulation method (Method 4), the value further decreased to 15.5 N, representing 50% of the maximum value measured with Method 1.

Yield Load: Determination of the yield load revealed similar trends. The highest values were measured using Method 1 (25.9 N), compared to Method 2 (18.2 N), which is 70% of the result from Method 1. The values further decreased with Methods 3 and 4, yielding 12 N. There is a significant difference between Method 1 and the other testing methods in both maximum and yield load (Figure 3c).

Stiffness: The stiffness of the vertebrae using Method 1 was 95 N/mm. Methods 2 and 4 produced very similar stiffness values (Method 2: 103.9 N/mm; Method 4: 93.4 N/mm). The mean stiffness value obtained using Method 3 was 127 N/mm, higher than any other method tested. Although there was no significant difference between all tested methods, it was notable that the standard deviations for Methods 3 and 4 were higher compared to Methods 1 and 2 (Figure 3d).

Young’s Modulus: The material constant of the vertebra obtained by Method 3 (232 N/mm) was significantly higher compared to the methods where the vertebral body remained intact (Method 1: 101 N/mm; Method 2: 130 N/mm). This increase was also evident in the stiffness, but not in the load to failure, suggesting that the displacement in Method 3 might be altered compared to the other methods. Interestingly, Method 4 (145 N/mm) did not show much alteration compared to the Young’s modulus obtained by Methods 1 and 2 (Figure 3e).

### 3.2. Determination of Relative Biomechanical Properties

**Figure 4:**
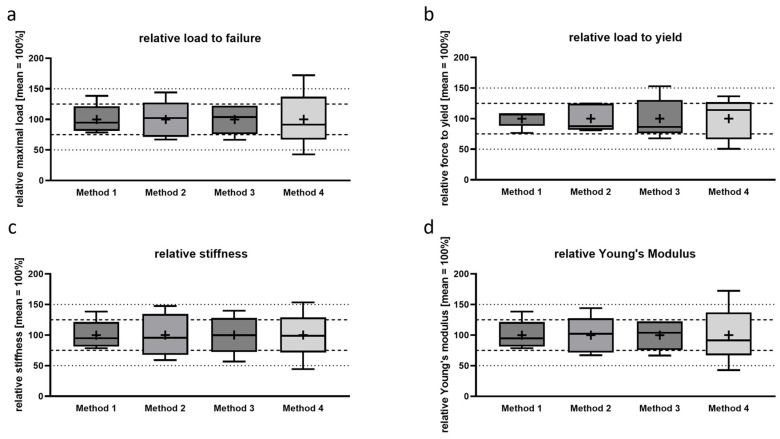
Load to failure (**a**), load to yield (**b**), stiffness (**c**) and Young’s modulus (**d**) normalized by setting each mean value (+) to 100%. The dashed line marks 25% and the dotted line 50% divergence from the mean.

To determine the relative deviation and reproducibility of each method, we calculated the experimental values in proportion to the mean, setting the mean value to 100%. We marked thresholds at 25% and 50% divergence from the mean to visualize abnormalities (Figure 4). In the box–whisker plot, the box represents the lower and upper quartiles of the values, encompassing the range where four of the five replicates are located. This conversion visualizes the reproducibility of the methods for given parameters.

For single-point parameters (load to failure and load to yield; Figure 4a,b), Method 1 showed the smallest deviation. Particularly for load to yield, the methods that kept the bone intact demonstrated good reproducibility compared to Methods 3 and 4. We investigated the slope of the force/displacement curve to present stiffness and calculate the Young’s modulus (Figure 4c,d). The overall reproducibility was lower for these parameters. It should be noted that, for these calculations, experimental values, such as displacement and force for stiffness and force, displacement, and diameter for Young’s modulus, were required, each with individual measurement errors. Despite these factors, Method 1 exhibited the lowest deviation across all the biomechanical parameters obtained.

## 4. Discussion

The aim of this study was to identify a valid and reproducible method to evaluate the biomechanical properties of the murine lumbar vertebral bodies. Our findings demonstrated that the vertebral body, characterized by a high cancellous-to-cortical bone ratio and active bone remodeling [7], is a suitable model for early detection of changes in bone quantity and quality. To determine the biomechanical properties of trabecular bone, compressive force is the closest testing method to the physiological circumstances [17], while bending and torsion forces are more suitable for testing cortical bones [6,18]. However, the lack of standardization in applying compressive force to vertebrae and measuring forces presents challenges.

In this study, we compared four compression testing methods to evaluate their validity and reproducibility. Our results indicated that Method 1, which involves minimal manipulation of the vertebral body, provided the most consistent and reproducible data. This method maintains the integrity of the vertebral body, ensuring that the force is applied uniformly across the entire structure.

Interestingly, our findings align with the study of Goetzen et al. (2005), who also tested vertebral bodies using an unmodified bone approach similar to Method 1 [8]. Their results, derived from 12-month-old C57BL/6 mice, showed consistency with our findings, albeit with a different age group. This consistency across different ages highlights the robustness of Method 1.

Tommasini et al. (2005) used a modified Method 2 with 15-month-old mice, involving grinding the cranial and caudal surfaces and fixing the vertebral body with a rod through the vertebral canal [12]. They reported a higher median load to failure (28.5 N) compared to our results (20.75 N) with 12-week-old mice. This discrepancy could be attributed to the age difference, as bone strength increases with age. In our previous studies with 16-month-old mice, we observed a similar median load to failure of 27 N.

Almeida et al. (2007) used Method 3, removing the vertebral arch and adjusting the inclined surfaces with a custom-made flexible bearing area [14]. Their studies with 8- and 16-week-old mice did not provide directly comparable data due to age differences. However, their approach highlights the variability introduced by extensive sample manipulation.

Akhter et al. (2001) employed a method similar to our Method 4 on 4-month-old C57BL/6 mice, finding results consistent with our study regarding load to failure, yield load, and stiffness [1]. Their findings support the notion that extensive manipulation can still yield valid results, though our study suggests Method 1 is more reliable overall.

Comparing all four methods, we observed that Methods 2–4, which involved partial vertebral body usage, resulted in lower absolute load to failure and yield load values. However, normalized parameters, like stiffness and Young’s modulus from Methods 2 and 4, were comparable to those of Method 1. Method 3 showed a significant divergence to Method 1 and 2, particularly for stiffness and Young’s modulus. Since load to failure and load to yield are in a similar range, it is likely that the displacement is different in this measurement, which is represented by the term ${∆l}_{f}-{∆l}_{0}$ in the calculation of stiffness (Formula (1)).

By normalizing the measured values to the mean, Method 1 exhibited the lowest deviation, while Method 4 showed the highest. Method 4′s extensive manipulation increased error risk during sample preparation. Although Method 3 produced consistent results among the samples, it likely contained a systematic error. As discussed above, we think that it is most likely that the displacement data was causing this difference. However, the distribution of the single measurements (Figure S1, stiffness, and Young’s modulus) did not suggest a systemic error.

This study has limitations, including the young age of the mice (12 weeks) and a small sample size (five per method). The age-related decline in trabeculae, common in older mice, was not considered, which could be relevant for Methods 3 and 4 that involve cortical damage during preparation. To calculate the Young’s modulus, we assume an elliptical shape for the area of the corpus vertebrae which is less complex to the actual shape. Method 1, which tests the whole vertebra, assumed a ring-like shape, which is, on the one hand, a different model and, on the other hand, an even stronger simplification of the actual shape. Despite these limitations, our results consistently support the validity of Method 1, especially for stiffness and Young’s modulus.

Overall, we recommend Method 1 for obtaining comparable data on the compression strength of murine lumbar vertebral bodies. Method 1, requiring the least sample manipulation, provided the most consistent data. Interestingly, Method 1 is the least frequently used in the literature, which we just found in a PhD thesis/congress report [8], with Methods 2 and 4 being published in peer reviewed articles. Nonetheless, we stick to our recommendation of Method 1, for Methods 2 and 4 require more processing steps, which increase the possibility of methodical errors. An instance when Method 2 could be preferred over Method 1 could be when the samples show extreme differences in size. This can occur when comparing old against young animals or male against female. Then, the more precise estimation of the vertebrae area can be beneficial as the Young’s module is more important under these circumstances.

Newer studies, like Zhong et al., 2025 [19] and Komrakova et al., 2024 [20], show the need of this research. Even though the L4 vertebrae were investigated, long bones, like femurs, are used for the biomechanical analyses, which are less suited to investigate osteoporotic bone degeneration [7,9,10].

This underscores the necessity for comparative studies like ours to standardize biomechanical testing procedures, enhancing reproducibility and reducing animal numbers.

## 5. Conclusions

In this study, we compared four different methods of preparing murine lumbar vertebrae to test their resistance towards compression in a material testing machine. With just embedding the whole caudal surface for even stand and applying force over the vertebral body and the transversal processes (Method 1), we identified the most reliable and reproducible compression testing procedure for lumbar vertebrae and could clearly demonstrate that the extent of manipulation to isolate parts of the bone or fix it has an impact on the outcome of the results. The more the bone was modified, the standard deviation of the values increased, pointing toward a decrease of reliability and reproducibility. Our results emphasize the need for standardized biomechanical testing procedures for bone in animal experiments in order to maximize significance and reproducibility and minimize animal numbers.

## Data Availability

The experimental data is available in the Supplements.

## Supplementary Materials

The following supporting information can be downloaded at: www.mdpi.com/article/10.3390/bioengineering12030273/s1, Figure S1: Scatter plot visualization of the biomechanical parameters obtained by compression testing the fourth lumbar vertebral body using the four given methods until failure. (a) Load to failure in N; (b) load to leave the elastic properties of the sample in N; (c) stiffness obtained by calculation to the slope in the force/displacement graph at the elastic increment in N/mm; (d) Young’s modulus normalizing the stiffness over the surface area to obtain the dimensionless material constant in N/mm.
