# Supplementary material for: Comparative Evaluation of Compression Testing Methods for Murine Lumbar Vertebral Bodies: Identifying Most Reliable and Reproducible Techniques for Assessing Compressive Strength
Source: Bioengineering (Basel). 2025 Mar 10;12(3):273. doi: 10.3390/bioengineering12030273 (PMC11939580; doi:10.3390/bioengineering12030273)
Supplement: Supplementary file 1 [file bioengineering-12-00273-s001.zip › bioengineering-3479565-supplementary.pdf]

bioengineering

Supplementary Materials

Comparative Evaluation of Compression Testing Methods for Murine Lumbar Vertebral Bodies: Identifying Most Reliable and Reproducible Techniques for Assessing Compressive Strength

Daniel Kronenberg [1,*], Britta Wieskoetter [2], Sarah Soeger [1], Heriburg Hidding [1], Melanie Timmen [1], Michael J. Raschke [3] and Richard Stange [1,2]

[1] Department of Regenerative Musculoskeletal Medicine, Institute of Musculoskeletal Medicine, University of Muenster, 48149 Muenster, Germany
[2] Department of Orthopaedics, Trauma, Hand and Reconstructive Surgery, University Hospital Münster, Marienhospital Steinfurt, 48565 Steinfurt, Germany
[3] Department of Trauma, Hand and Reconstructive Surgery, University Hospital Muenster, 48149 Muenster, Germany
* Correspondence: daniel.kronenberg@ukmuenster.de

 https://doi.org/10.3390/bioengineering12030273

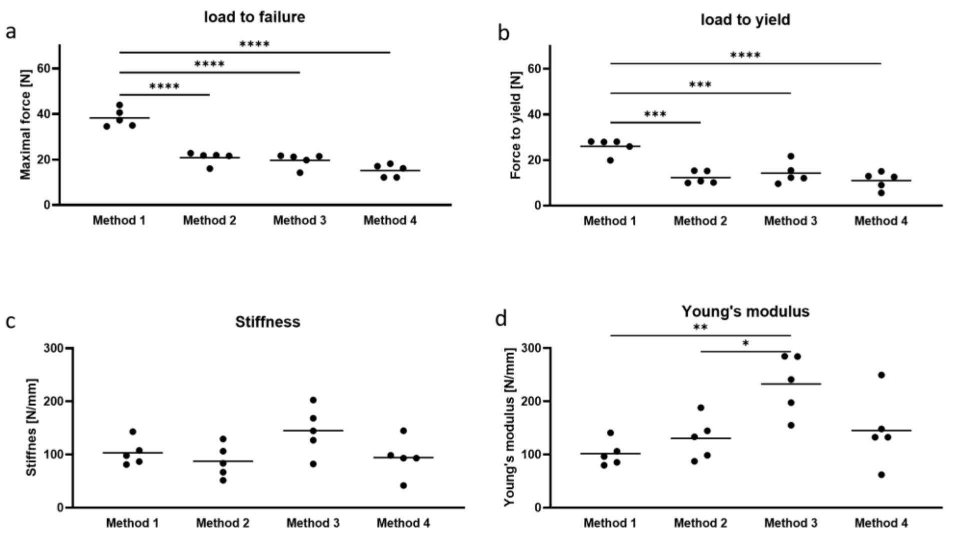

Figure S1. Scatter plot visualization of the biomechanical parameters obtained by compression testing the fourth lumbar vertebral body using the four given methods until failure. (**a**) Load to failure in N; (**b**) load to leave the elastic properties of the sample in N; (**c**) stiffness obtained by calculation to the slope in the force/displacement graph at the elastic increment in N/mm; (**d**) Young's modulus normalizing the stiffness over the surface area to obtain the dimensionless material constant in N/mm.
